# Assistive robotic hand with bi-directional soft actuator for hand impaired patients

**DOI:** 10.3389/fbioe.2023.1188996

**Published:** 2023-07-05

**Authors:** Kelvin H. L. Heung, Heng Li, Thomson. W. L. Wong, Shamay S. M. Ng

**Affiliations:** ^1^ Department of Building and Real Estate, Hong Kong Polytechnic University, Kowloon, Hong Kong SAR, China; ^2^ Department of Rehabilitation Sciences, Hong Kong Polytechnic University, Kowloon, Hong Kong SAR, China

**Keywords:** soft robotic hand, finite element method, pneumatic bending actuators, range-of-motion, force interaction

## Abstract

Soft wearable robotic hand can assist with hand function for the performance of activities of daily living (ADL). However, existing robotic hands lack a mathematical way to quantify the grip force generated for better controlling the grasp of objects during the performance of ADL. To address this issue, this article presents a soft wearable robotic hand with active control of finger flexion and extension through an elastomeric-based bi-directional soft actuator. This actuator bends and extends by pneumatic actuation at lower air pressure, and a flex sensor embedded inside the actuator measures the angles of the fingers in real-time. Analytical models are established to quantify the kinematic and tip force for gripping of the actuator in terms of the relationship between the input pressure and the bending angle, as well as the output force, and are validated experimentally and by the finite element method. Furthermore, the ability of the soft robotic hand to grasp objects is validated with and without being worn on a human hand. The robotic hand facilitates hand opening and closing by the wearer and successfully assists with grasping objects with sufficient force for ADL-related tasks, and the grip force provided by the actuator is further estimated by the analytical models on two healthy subjects. Results suggest the possibility of the soft robotic hand in providing controllable grip strength in rehabilitation and ADL assistance.

## Introduction

Soft robotics is an emerging field of robotics that involves the development and use of robots made of soft and flexible materials, such as silicone, rubber, and plastics (Marchese et al., 2015; Rus and Tolley, 2015; Jin et al., 2022). Unlike traditional mechanical robots that are typically made of rigid materials and have limited flexibility, soft robots are designed to mimic the movements and functions of natural organisms, such as octopuses, worms, and even human muscles (Heung et al., 2016; Das et al., 2023). In particular, soft robotic hands have several advantages over traditional mechanical robotic hands in terms of increased compliance, lower inherent stiffness, lighter weight, lower cost, and ability to provide customizable motion (Cappello et al., 2018a; Heung et al., 2019a; Mohammadi et al., 2020; Tang et al., 2022; Das et al., 2023). Several pioneering works have been accomplished by Cappello et al. (2018a), Heung et al. (2019a), and Yap et al. (2017a), which adopted pneumatic powered soft actuators to control the flexion and extension of fingers in healthy or subjects with impaired hand function. The actuators used in the soft robotic hands consisted of two sets of chambers which were able to generate both Range-of-Motion (ROM) and torque in two different directions. By applying pressure to the upper bending chamber, the actuator was able to bend towards the bottom extension chamber, and conversely, to facilitate the respective movements of flexion and extension.

Previous studies have effectively modeled the performance of soft actuators upon pressurization (Polygerinos et al., 2015; Connolly et al., 2017; Ali et al., 2022; Namdar Ghalati et al., 2022; Yu et al., 2022). Common practice for characterizing a soft actuator is to measure the angle-pressure and force-pressure relationships, which are widely accepted parameters for people objectively comparing the actuation performance of different actuator designs. Several studies have further attempted to derive an equation that relates the input pressure to both the Range-of-Motion (ROM) and contact force of soft actuators (Polygerinos et al., 2015; Namdar Ghalati et al., 2022). However, this has been challenging due to the nonlinear feedback generated in response to pressurized fluid. The modeling of the bi-directional soft actuator plays a crucial role in controlling the performance of grasping through pressurization. This is particularly important because the force and ROM required to grasp different daily objects varies based on factors such as their size, weight, and shape. The development of a mathematical model of the bi-directional soft actuator is essential for systematically understanding the performance of grasp of objects upon pressurization and serving as guidance regulating the input pressure to produce the optimal ROM and output force required for grasping these objects effectively.

Implementing a suitable sensing system is crucial to be integrated with the mathematical model of the bi-directional soft actuators for optimal control of grasping performance with soft robotic hands. Optical-fiber-based sensing methods, such as macro-bend optical stretch sensors (Sareh et al., 2015) and Fiber Bragg Grating (FBG) (Xu et al., 2017; Yang et al., 2020), have been utilized to detect bending, elongation, compression, and output force of deformable soft robotic actuators. However, these methods require an external electromagnetic tracking system to monitor actuation performance, which can be very accurate but also expensive and impractical for wearable applications. In contrast, Hall-effect sensors can be used as proximity sensors from a magnetic source and provide accurate information on module curvature when embedded within the soft actuator body. Despite their high accuracy, interference from other electromagnetic sources near the actuator can limit the effectiveness of these sensors.

Embedded sensors that can withstand large deformations without affecting the actuator’s high compliance are the most appropriate sensing method for the measurement of bending angle and output force with wearable robotic devices. Electronic circuits of such sensors can be printed on soft, stretchable, foldable, and biocompatible materials, such as silicone rubber (Majidi et al., 2011; Martirosyan and Kalani, 2011; Zhang et al., 2021), which conforms to the bi-directional soft actuator deformation without interfering with the natural mechanics of motion. Flexible angle sensors that produce a change in electrical resistance when bent have been introduced for wearable applications due to their compactness, low-cost, and lightweight solutions for soft robotics. These sensors are often integrated onto wearable robotic devices, such as soft robotic hands, for the measurement of actuator angles during pressurization (Simone and Kamper, 2005). However, there are limited suitable force sensors for measuring the contact force of soft robotic hands. The only commercial force resistive sensors (FSRs) available are commonly used as a trigger for device on/off and not for the measurement of contact force. FSRs suffer from several limitations, including hysteresis, which can cause differences in readings depending on whether the force is increasing or decreasing, and drift, which can result in long-term variations in output readings. Additionally, the accuracy of FSRs can be affected by the contact area and force distribution, especially when measuring uneven surfaces. Therefore, while flexible angle sensors provide a better method for angle measurement in soft actuators, there is still a need for an optimal approach for output force characterization with soft actuators.

**FIGURE 1:**
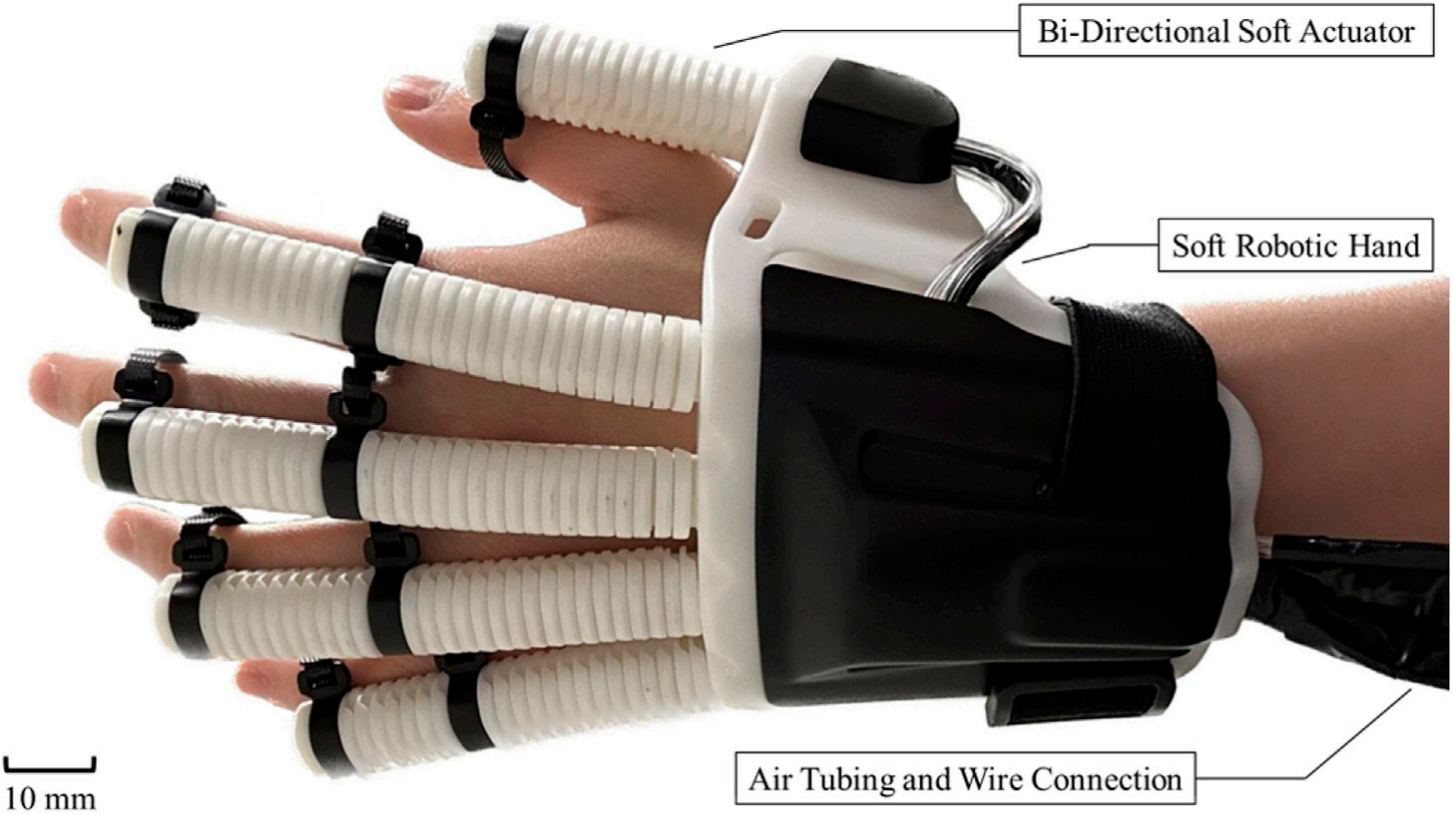
Prototype of the soft robotic hand with the bi-directional soft actuator.

Previously, we also designed the soft pneumatic actuator that a stiff metal spring was embedded with the elastomeric cavities with the purpose of increasing the passive extension force when extending the finger joints (Heung et al., 2019a; Heung et al., 2019b). The actuator featured a composite design that incorporated a metal spring capable of facilitating both flexion and extension within a single unit. Flexion and extension of the actuator were controlled by pressurization and depressurization, respectively. Upon pressurization, the actuator’s fiber wrapping suppressed radial expansion and permitted axial elongation along the actuator. At the bottom of the actuator, the metal spring constrained axial elongation, enabling the upper section of the actuator to elongate and generate the bending movement. Upon depressurization, the spring provided an assistive bending moment for finger extension to the initial position. However, this approach significantly hindered the bending ability of soft actuator, as well as joint flexion movements due to the high stiffness of the metal spring (Shi et al., 2021). Therefore, this study details the new design of an optimal bi-directional soft actuator, which also allows for active control of both bending and extension with less input pressure required comparing with our previous design of Soft-Elastic Composite Actuator (SECA) (Heung et al., 2019a), while maintaining a size comparable to a normal finger. The actuators are embedded with flexible angle sensors and extension of the actuator is limited by its inherent mechanical structure, preventing over-extension beyond its resting position when uninflated. A mathematical model is presented that describes the bending angles and output force of the actuator based on the input pressures in the two respective actuation cavities for bending and extension. Additionally, a portable soft robotic hand system prototype integrating multiple actuators is introduced (Figure 1). Experimental results are presented, including bending angles and output force measurements, as well as validation through finite element method (FEM) and analytical results. Lastly, the capability of the device to assist with object manipulation through suitable bending angles and output torque is further demonstrated with and without being worn on human hand. Two healthy subjects are recruited for evaluating the mathematical model of estimating the grip force provided by the passive assistance generated by the bi-directional soft actuator upon pressurization.

## Soft robotic hand system

### Bi-directional actuator design

In earlier versions of soft robotic hands, the actuators were responsible for flexing the distal interphalangeal (DIP), proximal interphalangeal (PIP), and metacarpophalangeal (MCP) joints. However, it has been found that the DIP joint plays a limited role of 15% in functional gripping (bovic and Bowers, 1994). Additionally, to facilitate sensory feedback while interacting with the surroundings, it is important to provide the user with a sense of touch at the fingertips (Tong et al., 2010). Therefore, our bi-directional soft actuator does not cover the DIP joint.

**FIGURE 2:**
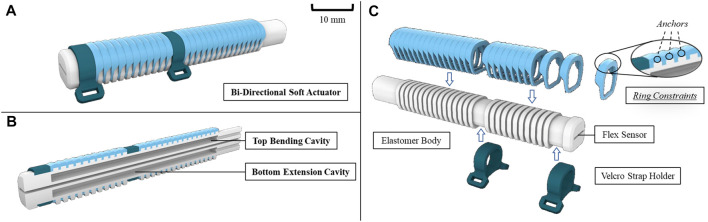
**(A)** General design of the bi-directional soft actuator, **(B)** cross-section area view showing the separated actuation cavities, **(C)** exploded view showing the ring constraints, elastomer body embedded with flexible angle sensor, and the Velcro holder for securing on the hand.

Our latest bi-directional soft actuator inherits the advantages of being lightweight, safe, and having a lower inherent impedance compared to any electric counterpart (Figure 2A). In contrast to our previous design, which incorporated a stiff metal spring, our current design offers a larger ROM. The actuator comprises two internal cavity layers for pressurized fluid application (Figure 2B). One cavity controls flexion, while the other controls extension. Upon pressurizing the top cavity, the actuator flexes towards the bottom cavity, and *vice versa* upon pressurizing the bottom cavity. Therefore, pneumatic sources can effectively control flexion and extension, generating a much larger ROM. To attach the bi-directional soft actuator onto the hand, we use Velcro Strap holders. We also wrap rigid ring constraints around the outer surface of the actuator to eliminate any irregular expansion of cavities, and hence to facilitate actuator flexion and extension upon injection of pressurized fluid (Figure 2C). Additionally, anchors in the ring constraints around the top surface of the actuator body further restrict over-extension beyond the top surface when the bottom cavity is pressurized. Finally, we placed a commercially available 4.5 inches angle sensor (Flexpoint Sensor System, Draper, UT, United States) in the actuator to measure its bending angles for flexion and extension (Wang et al., 2020).

### Device fabrication

**FIGURE 3:**
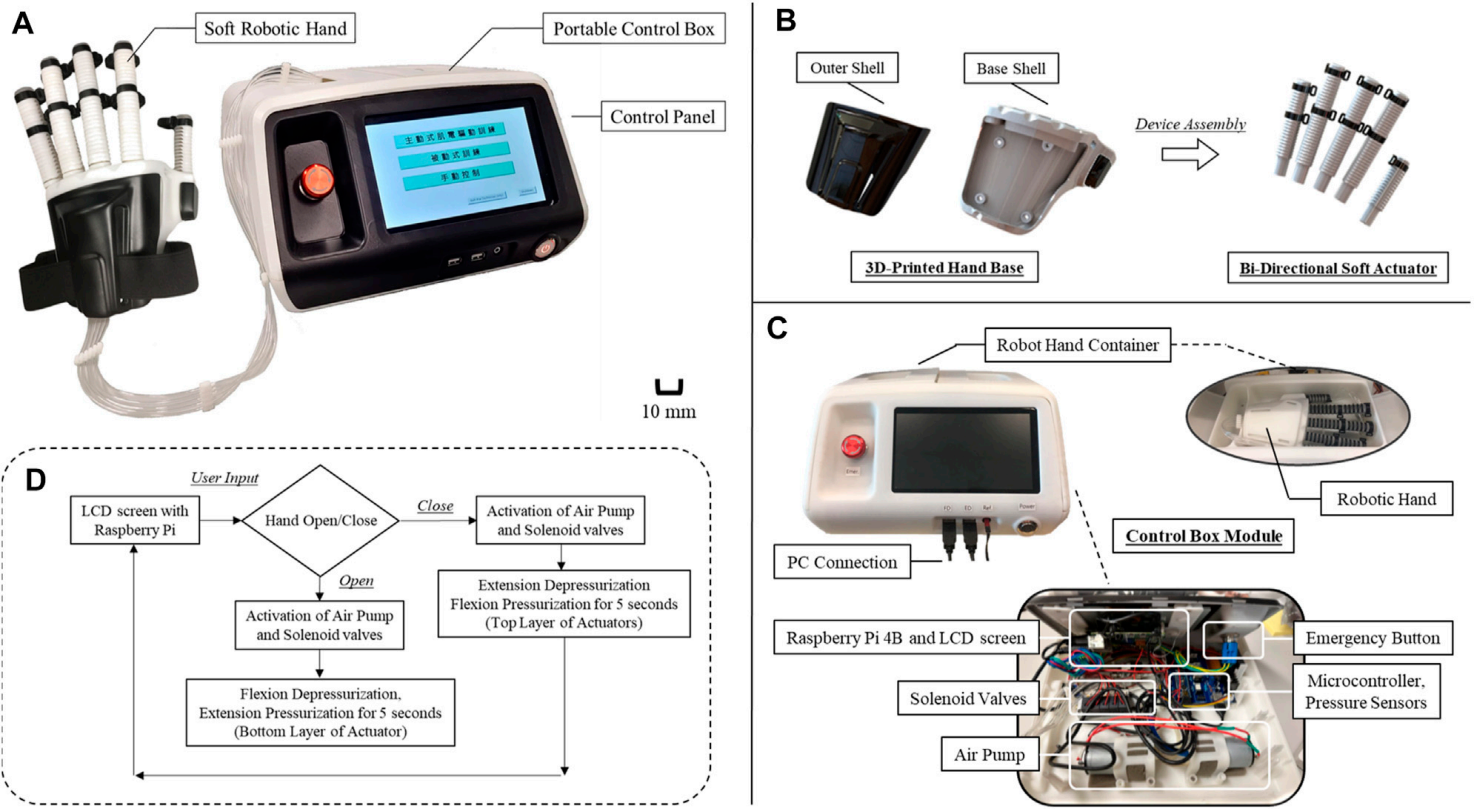
**(A)** Complete setup of the robotic hand system, **(B)** exploded view of the robotic hand showing features of multiple bi-directional soft actuators and plastic hand base for securing the actuators, **(C)** design structure of the control box, **(D)** control logic of the robotic hand for facilitating hand closing and opening movement.

The robotic hand system includes the robotic hand and the tethered control box (Figure 3A). For the actuator, the virtual lost wax casting method (Heung et al., 2016) is used. Unlike the conventional lost wax casting method, no wax is used in this process as the mold is 3D printed. The detailed fabrication process of soft actuators is available in (Soft Robotic Toolkit). It involves printing the mold, filling it with a mixture of silicone rubber, curing and demolding the actuator. The pliable silicone rubber used in the actuator is Dragon Skin 30 (Smooth-On, Inc., PA, United States) that provides sufficient elongation (up to 364%) and hardness (Shore A 30) to support large deformations and prevent material failure and rupture in the bended state (Dragon Skin Series). For the ring constraints and the hand base, they are 3D printed by Polyethylene (PE). Multiple actuators that are embedded with ring constraints are further secured onto the fingers via the hand base that accommodates the size of human hand. Two black Velcro straps are used to tighten the proximal interphalangeal (PIP) and metacarpophalangeal (MCP) joints onto individual finger as well (Figure 3B). The bi-directional soft actuator is 12 mm wide, 12 mm high, and 65 mm (thumb), 85 mm (small finger), or 105 mm (three fingers). The size of the robotic hand is 17 cm (length) × 10 cm (width) × 3 cm (height), and the weight of the actuator and robotic hand are 19 g and 176 g, respectively.

### Portable control box hardware

The control box inside includes all standard pneumatic components, including air pump (BTC Diaphragm Pump, Parker Hannifin Corporation, United States), solenoid valves (ITV2091-21N2BS5, SMC, Japan), air tubes, pressure sensors (XGZP6847, C-LinkTech, Korea), and LCD touchscreen [7 inches, 800 × 480 pixels (RGB), Raspberry Pi, United Kingdom], for controlling the pressure supplied to the actuator. Solenoid valves and air sources are either turned on or off by the control signal from Arduino Mega 2,560 for the regulation of pressure supplied to the actuator (Figure 3C). PWM signals from the Arduino are connected to the voltage switch IRF530N for controlling high voltage devices using Arduino. Pressure sensors are used to monitor the pressure supplied to the soft actuator. The control of Arduino Mega 2,560 that regulates all the signal processing requires a Raspberry Pi 4B. The LCD touchscreen presents the control panel to users, allowing them to control the system without connecting to computers (Figure 3D). The panel allows users to manually select the model of either hand closing or opening. Upon selecting the hand closing option, bottom layer of the actuator will be depressurized, and the top layer will be pressurized for 5 s for inflation of the chamber, and *vice versa* in case of choosing hand opening. Eventually, the Raspberry Pi records the joint angles measured and sends out control signals to the solenoid valves for controlling the soft actuators, as well as to identify the output force with its mathematical model.

On the top of the control box, there also exists a container for the storage of the robotic hand. An emergency button is installed next to the control panel to immediately depressurize the actuators in case of emergency termination of usage. The connection ports next to the power button allow users to transfer the stored data, e.g., joint angles, in the Raspberry Pi 4B back to computers (Figure 3C). Eventually, the control box is 3D printed and designed to be portable, which is 30 cm (length) × 30 cm (width) × 21 cm (height), and the weight is 1.7 kg.

## Bi-directional soft actuator modeling

**FIGURE 4:**
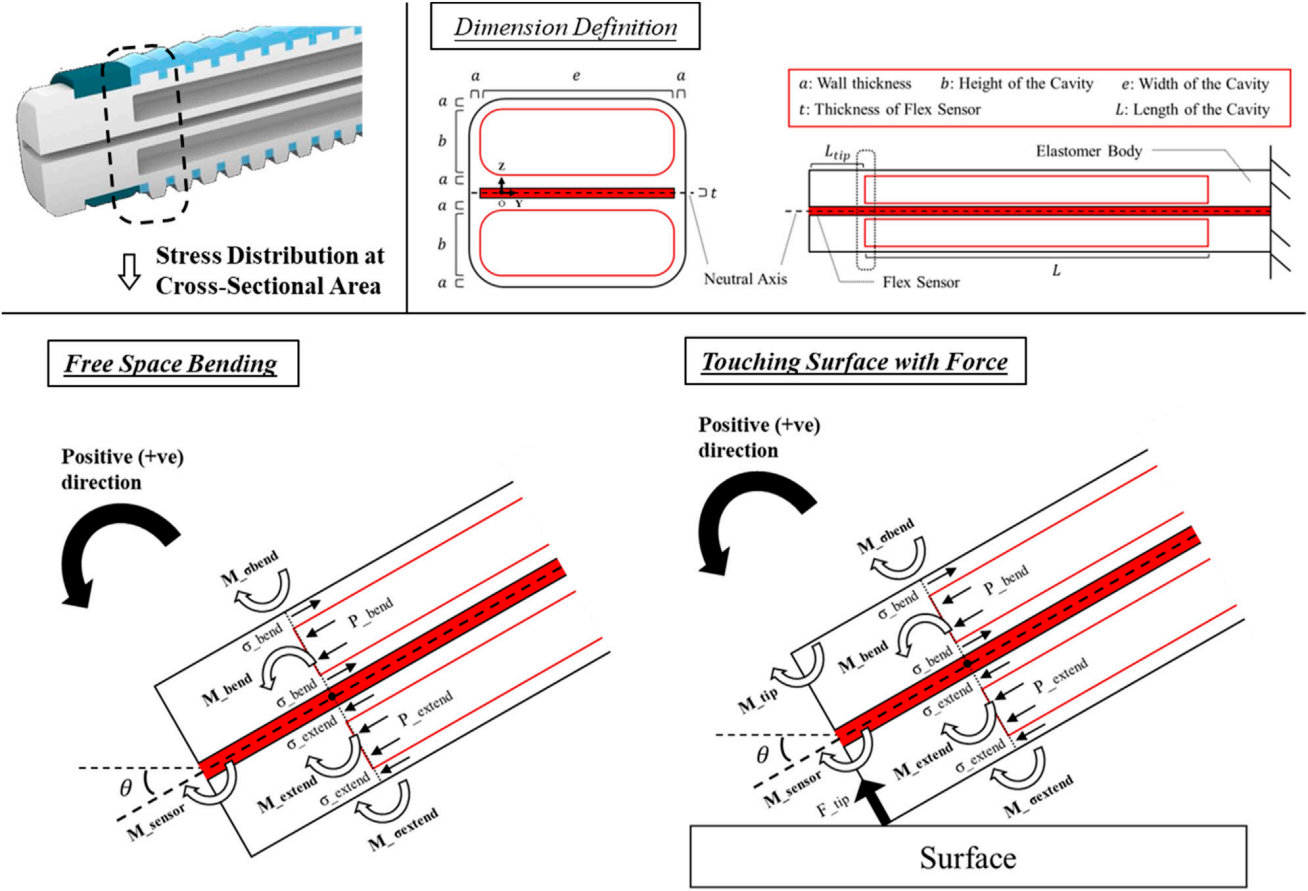
Side view defining the actuator dimension and illustrating all the torque generated around the fulcrum O in a bending state at the tip of the bi-directional soft actuator during free space bending and the contact with objects.

A mathematical model has been developed and presented which illustrates the correlation between the input pressures and the bending angle as well as the output force of the actuator. This model is static in nature. Furthermore, the model considers the impact of the resistance created by the flex sensor (Wang et al., 2017) and elastomer body, as well as the bending moment that arises from fluid injection into the cavities, in order to provide an accurate representation of the actuator (Figure 4).

### Bending moment arose from fluid injection

When pressure is applied, the actuator undergoes bending motion which is dependent on the pressure levels inside the two separate cavities. Assuming that the cavities are rectangular in shape and do not experience any cross-sectional deformation (Heung et al., 2020; Raeisinezhad et al., 2021), the bending moment resulting from the pressure applied to each cavity can be determined by.

(1) $M_{bend}$ , moment for actuator bending.

Mbend=∫a+t2b+a+t2e∙Pbend∙z∙dz (1)

(2) $M_{extend}$ , moment for actuator extension.

Mextend=∫−b+a+t2−a+t2e∙Pextend∙z∙dz (2)

where a is the wall thickness, b is the internal rectangular height, e is the internal chamber width, t is the thickness of flex sensor, L is the internal chamber length, P is the input pressure, and dz represents the differential height element in z -direction.

### Resistance created by the elastomer body

The bending of the actuator causes the elastomer body and flex sensor to resist the bending deformation and create a bending moment in the opposite direction to the bending itself, which can be determined by

(3) $M_{\sigma bend}$ and $M_{\sigma extend}$ , elastomeric resistive moment.

The bi-directional soft actuator is constructed using Dragon Skin 30 silicone rubber. This material can be described using an Ogden first order hyper-elastic model (Sparks et al., 2015). The strain energy of the material is expressed as follows

$$W\left(\lambda_{1},\lambda_{2},\lambda_{3}\right)=\frac{2\mu }{{\alpha_{1}}^{2}}\left({\lambda_{1}}^{\alpha_{1}}+{\lambda_{2}}^{\alpha_{1}}+{\lambda_{3}}^{\alpha_{1}}-3\right)$$ (3)

where the material coefficient $\alpha_{1}$ is the strain hardening exponent, and μ is the small strain shear modulus. Details of determining the internal stress $\sigma_{bend}$ and $\sigma_{extend}$ that oppose the bending deformation of actuator based on the Ogden material model in Eq. 4 has already been addressed in (Heung et al., 2019a), which can be expressed by

$$\begin{array}{c} \sigma_{bend}=\sigma_{extend}\\ {=\mu }_{1}\left(\lambda^{\alpha_{1}}-\lambda^{{-\alpha }_{1}}\right) \end{array}$$ (4)

that

$$\lambda =\frac{\theta z+L}{L},\mu_{1}=\frac{2\mu }{\alpha_{1}}$$ (5)

where λ is the axial stretch along the actuator’s length in x -direction, and therefore

Mσbend=∫t2a+t2e∙μ1λα1−λ−α1∙z∙dz+2∫t2b+2a+t2a∙μ1λα1−λ−α1∙z∙dz+∫b+a+t2b+2a+t2e∙μ1λα1−λ−α1∙z∙dz (6)

and

Mσextend=∫−a+t2−t2e∙μ1λα1−λ−α1∙z∙dz+2∫−b+2a+t2−t2a∙μ1λα1−λ−α1∙z∙dz+∫−b+2a+t2−b+a+t2e∙μ1λα1−λ−α1∙z∙dz (7)

(4) $M_{sensor}$ , moment of the flex sensor.

$$M_{sensor}=\frac{EI}{L_{sensor}}∙\theta$$ (8)

where EI is the flexural rigidity ( E is the elastic modulus, I is the second moment of area) of the sensor, $L_{sensor}$ is the length of the sensor, and θ is the bending angle of the bi-directional soft actuator. As part of this investigation, the flex sensor has been represented by a PE plastic film. The sensor has a width of 6.35 mm and a length of 114.3 mm (Spark Fun). Its elastic modulus and thickness are assumed to be 1 GPa and 1 mm.

### Contact force estimation

When the proximal tip of the actuator is securely mounted, it will apply a force $F_{tip}$ upon contact of its distal tip with an external object (Wang et al., 2017). This force is perpendicular to the bottom layer to maintain a constant bending moment arm of $L_{tip}$ , which is the length of the actuator tip, relative to the fulcrum O . The exerted moment is given by

$$M_{tip}=F_{tip}∙L_{tip}$$ (9)

Note that we implicitly assume that the force interaction happens at the end of the actuator, and we do not account for deformation along the actuator due to force exertion (Wang et al., 2017). Eventually, the response of bending angle to the input pressure can be found by the moment equilibrium achieved around the fulcrum O in bending. The integrals of moment equilibrium can only be solved numerically (Wang et al., 2017; Heung et al., 2019a; Heung et al., 2020), which is expressed by

a) Free Bending

$$M_{bend}+M_{extend}={M_{\sigma bend}-M}_{\sigma extend}+M_{sensor}$$ (10)

which

$$\left(P_{bend}-P_{extend}\right)=\frac{2}{eb\left(2a+t+b\right)}\left(\frac{EI\theta }{L_{sensor}}+f\left(\theta \right)\right)$$ (11)

and.

$$f\left(\theta \right)={M_{\sigma bend}-M}_{\sigma extend}$$ (12)

b) Touching Objects with Contact Force

$$M_{bend}+M_{extend}=M_{\sigma bend}-M_{\sigma extend}+M_{sensor}+M_{tip}$$ (13)

which

$$\begin{array}{c} \left(P_{bend}-P_{extend}\right)=\frac{2}{eb\left(2a+t+b\right)}\\ ∙\left(\frac{EI\theta }{L_{sensor}}+f\left(\theta \right)+F_{tip}L_{tip}\right) \end{array}$$ (14)

and

$$f\left(\theta \right)={M_{\sigma bend}-M}_{\sigma extend}$$ (15)

### Finite element method (FEM) modeling

**FIGURE 5:**
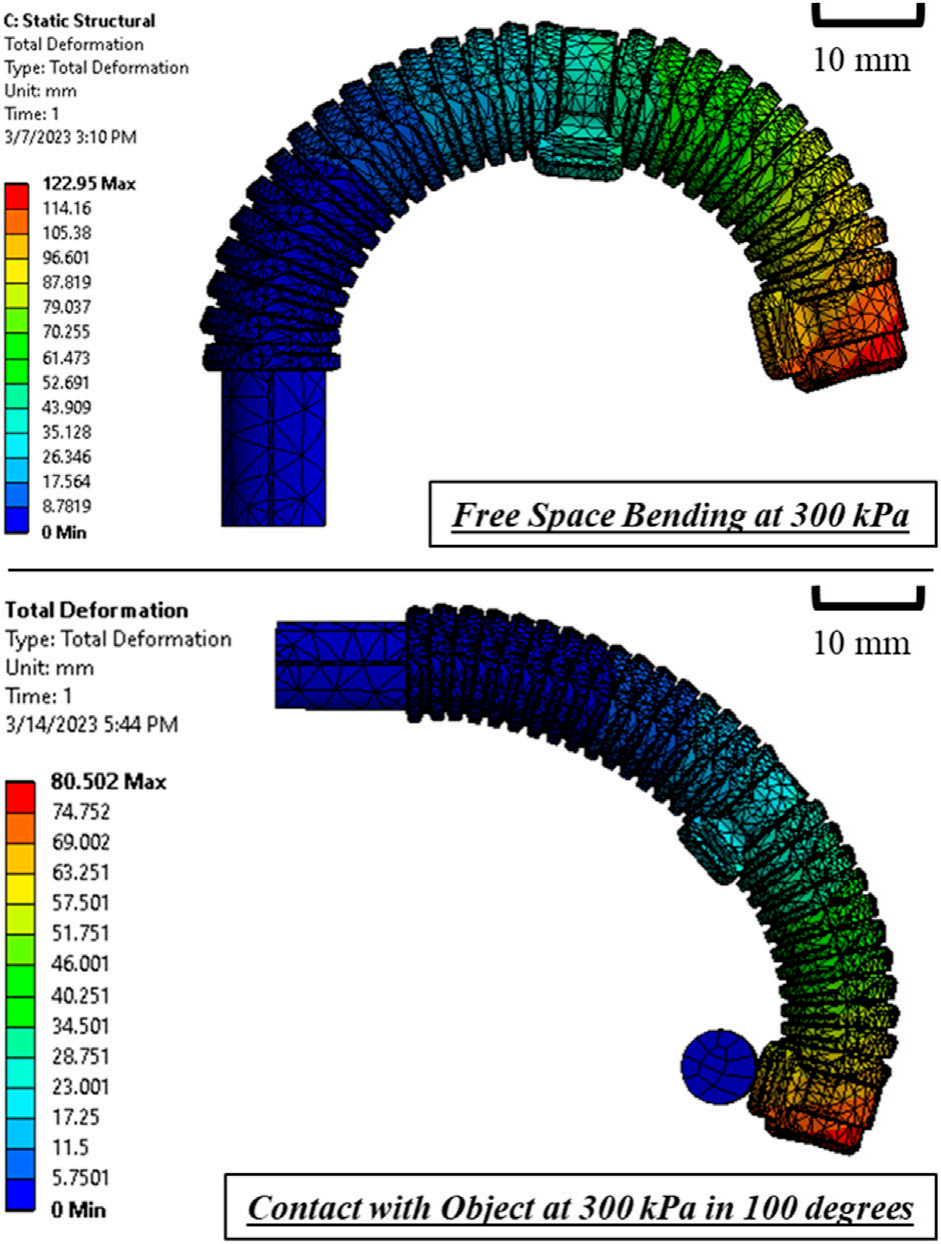
FEM-simulated bending of the bi-directional soft actuator at pressure input of 300 kPa.

We utilized ANSYS Workbench 15 to establish a 3D FEM model for the bi-direction soft actuator (Figure 5). The model was subjected to a Static Structural analysis to determine the bending angle and output force of the actuator under various input pressures. The settings of model were previously reported in our work (Heung et al., 2019a), with only one simplification made, which involved neglecting the pressure inlets and directly applying pressure to the internal chamber walls. To ensure accurate results, 3D 10-Node tetrahedral structural solid elements (ANSYS element type SOLID187) were used for both the elastomeric actuator body and the ring constraints, while 3D 20-Node structural solid elements (ANSYS element type SOLID186) were used for the thin film flex sensor. An Ogden first order hyper-elastic model with the coefficients $\mu_{1}$ = 75,449 Pa and $\alpha_{1}$ = 5.836 is used to model Dragon Skin 30 as it matched well with experiments (Sparks et al., 2015). For the ring constraints and flex sensor (assumed as Polyethylene), the material properties are directly obtained from ANSYS Engineering Data Sources.

## Experimental evaluation

### Measurement setup

**FIGURE 6:**
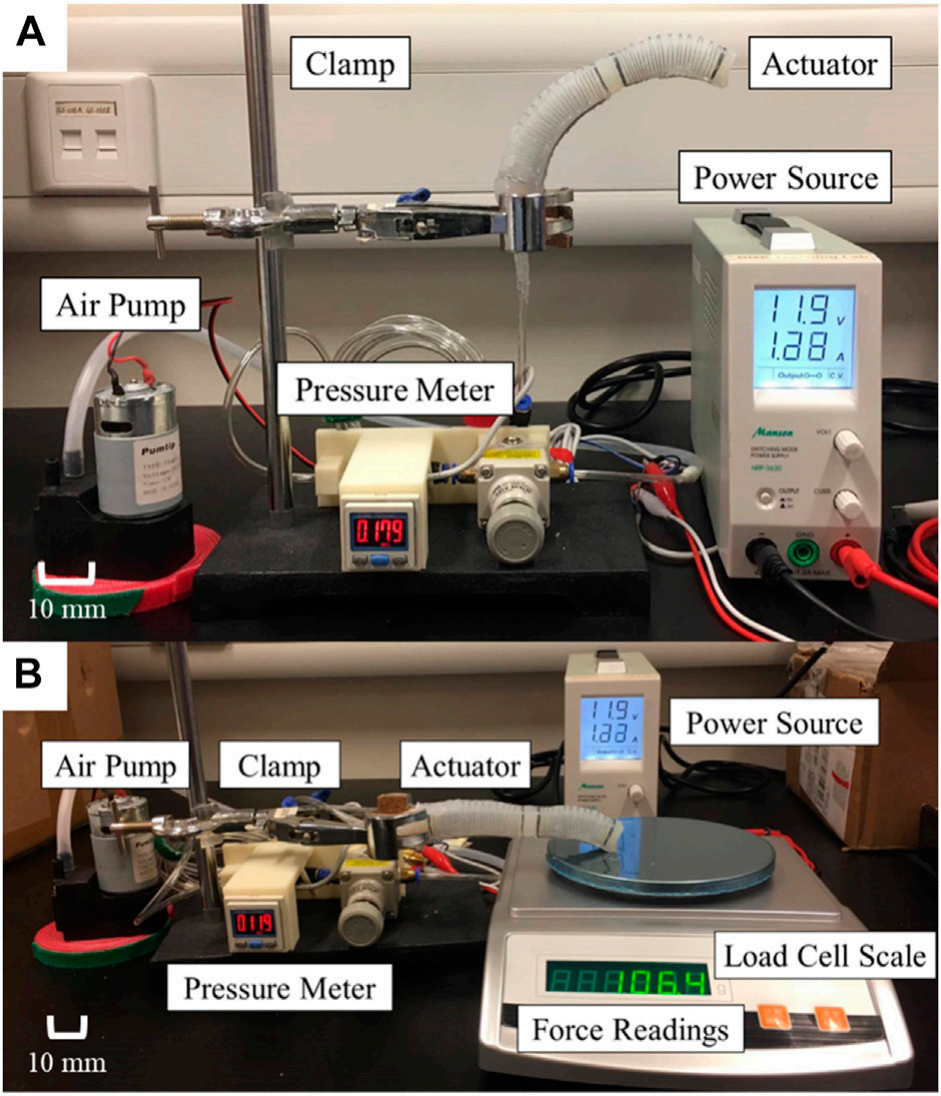
Example setup for actuator characterization of **(A)** free space bending and **(B)** contact force measurement.

Our previous studies (Heung et al., 2019a; Heung et al., 2019b; Heung et al., 2020; Tang et al., 2022) have already described the architecture of the pneumatic control setup used to measure bending angle and output force (Figure 6). The bi-directional soft actuator is supplied with air pressure from an air pump (BTC Diaphragm Pump, Parker Hannifin Corporation, Ohio, United States), which is controlled by a pressure meter [ZSE20C(F), SMC Pneumatic, Tokyo, Japan] and a pressure regulator (IR2020-02BG, SMC Pneumatic, Tokyo, Japan). The pressure regulator can be manually adjusted to control the air pressure supplied to the actuator, and the pressure value is displayed on the pressure sensor’s screen. A 12V voltage source is also connected to the system for operation.

**FIGURE 7:**
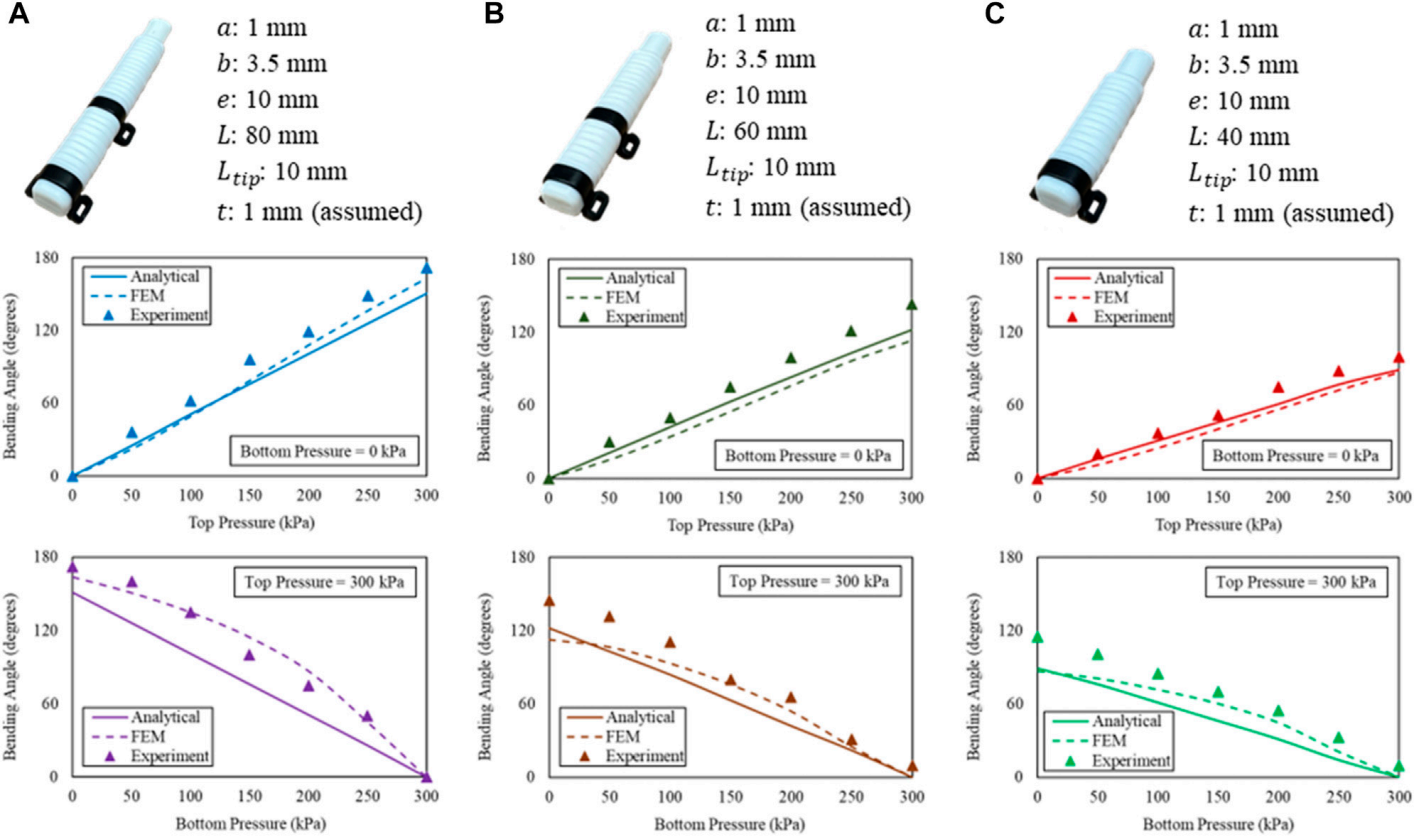
Pressure-angle relationship of the bi-directional soft actuator corresponding to **(A)** index, middle, ring fingers, **(B)** small finger, and **(C)** thumb.

**FIGURE 8:**
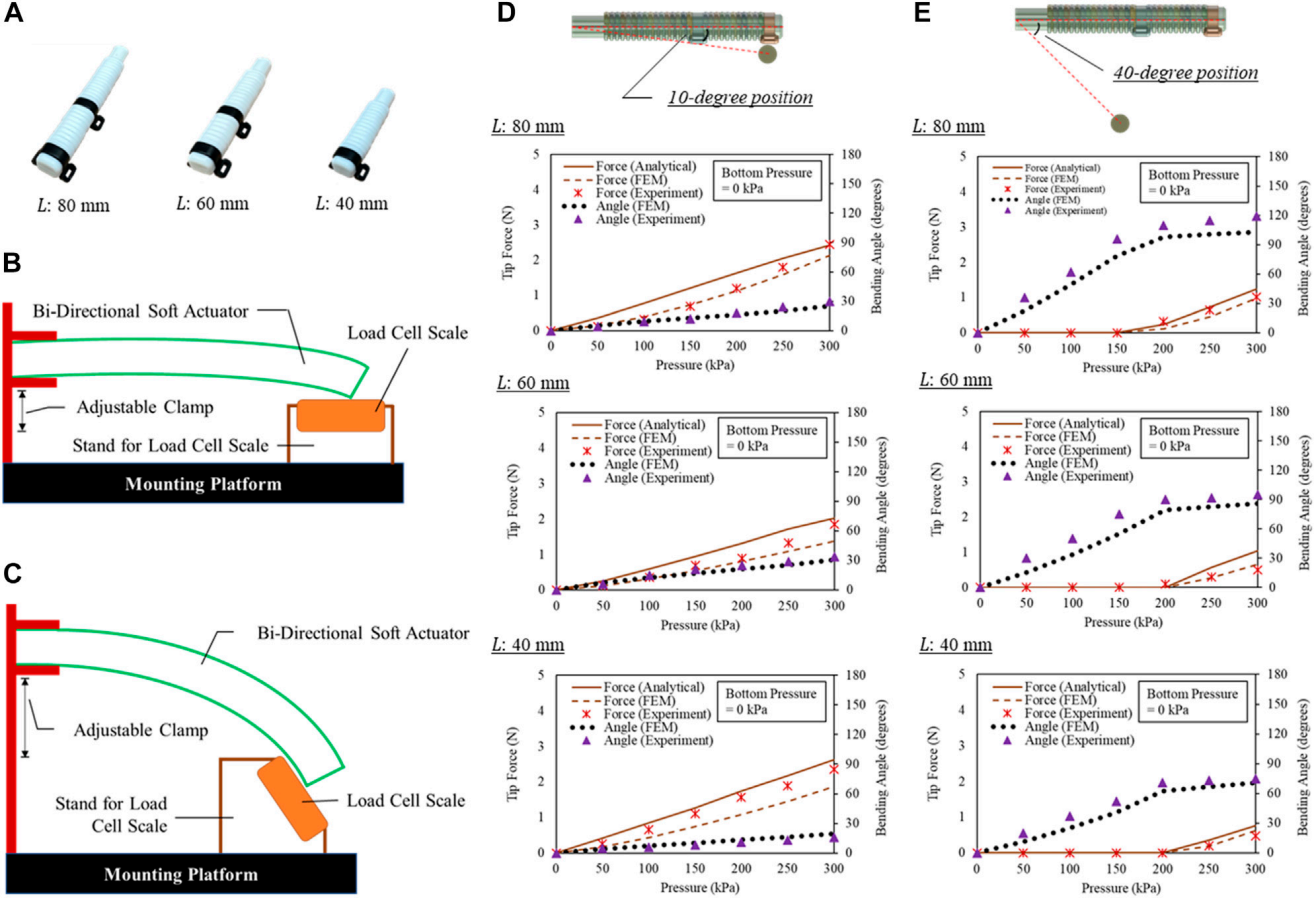
**(A)** Same actuators in Figure 7 were applied in force estimation, output force measurement at **(B)** 10-degree and **(C)** 40-degree angular positions with respect to the proximal ends of the actuators, **(D)** pressure-force relationship of the actuators at **(D)** 10-degree and **(E)** 40-degree angular positions.

In the experiments, three sets of actuators were evaluated (Figures 7A–C). They were tested for the bending angles in free space, contact forces at 10°, and 40°, respectively. The same equipment settings were applied for both free space bending and output force measurement. The proximal end of the actuator that connected with air tube was clamped. The bending angle of actuator was captured and measured using image analysis software (ImageJ, National Institute of Health, Maryland, United States). Simultaneously, the output force was measured using a compression load cell (FC22, Measurement Specialties, United States) placed on a stand with adjustable slope (Figures 8B, C). When pressurized, the load cell impedes the bending motion, and the desired generated tip force of the actuator could be captured simultaneously.

### Bending angle in free space measurement

The top bending cavity and bottom extension cavity were subjected to pressures ranging from 0 kPa to 300 kPa, with increments of 50 kPa. The resulting bending angles were then compared with those predicted by the analytical models and FEM simulations. The flex sensor minimized the effect of gravity on the bending angles, allowing for accurate measurements. The maximum input pressure for the actuator was limited to 300 kPa. The experimental results aligned well with both analytical models and FEM simulations, demonstrating that the bending angles increased with the length of the actuator at the same input pressure level. For instance, the actuator corresponding to the index, middle, and ring fingers achieved a bending angle of 172°, while the FEM simulation predicted 164° and the analytical model predicted 151°. The maximum difference of 34° between the analytical model and experimental results was observed in the bending of actuator corresponding to the three fingers at 300 kPa when the extension cavity was also pressurized to 100 kPa. It is important to note that our bi-directional soft actuator and the SECA in previous studies (Heung et al., 2019a; Heung et al., 2020; Shi et al., 2021; Tang et al., 2022) achieve actuator extension through different methods, including pneumatic pressurization and stiff metal springs, respectively. However, the bi-directional soft actuator might outperform the SECA by offering a larger ROM with less input pressure while maintaining control over the actuator extension. SECA cannot generate a larger ROM without increasing input pressure, which reduces its lifespan and leads to rupture. Therefore, for the design of a soft robotic hand, the durability of the actuator is a crucial consideration, and the bi-directional soft actuator is recommended for controlling finger movement.

### Force estimation based on actuator modeling

The top bending cavity was subjected to pressures ranging from 0 kPa to 300 kPa, with increments of 50 kPa (Figures 8A–E). The resulting output force was then compared with those predicted by the analytical models and FEM simulations as well. It is important to record the bending angles of the actuators during measurement, as the actuators will continue to bend (bulge) when subjected to increasing input pressure while in contact with objects. This bulging will affect the output force and should be taken into consideration.

Furthermore, in order to calculate the output force of the device using analytical models and Eqs 10, 13, it is necessary to determine the bending angles of the actuators. To estimate the output force at the tip of the device, the bending angles obtained from FEM simulations were used in the models to calculate the analytical force when the actuators were under pressure and impeded by objects placed at 10-degree and 40-degree angular positions, respectively.

### 10-degree angular position with respect to proximal end

The actuators experienced bulging when they contacted objects at bending angles of 30° (actuator representing the three fingers, FEM of 24.9°), 34° (actuator representing the small finger, FEM of 30.9°), and 16° (actuator representing the thumb, FEM of 19.2°), respectively, when a pressure of 300 kPa was applied. The measured output force was 2.45 N (actuator representing the three fingers, FEM of 2.13 N, Analytical of 2.43 N), 1.85 N (actuator representing the three fingers, FEM of 1.38 N, Analytical of 2.03 N), and 2.36 N (actuator representing the three fingers, FEM of 1.87 N, Analytical of 2.63 N), respectively. The maximum difference of 0.52 N between the analytical and experimental results was observed on the actuator corresponding to the three fingers when pressurized to 150 kPa in 10-degree position. No wall rupture or air leakage was observed during the tip force measurement.

### 50-degree angular position with respect to proximal end

The actuators experienced insignificant bulging when they contacted objects at bending angles of 119° (actuator representing the three fingers, FEM of 103.9°), 95° (actuator representing the small finger, FEM of 85.9°), and 75° (actuator representing the thumb, FEM of 70.5°), respectively, when a pressure of 300 kPa was applied. The measured output force was 1.02 N (actuator representing the three fingers, FEM of 0.97 N, Analytical of 1.24 N), 0.50 N (actuator representing the three fingers, FEM of 0.66 N, Analytical of 1.05 N), and 0.48 N (actuator representing the three fingers, FEM of 0.62 N, Analytical of 0.76 N), respectively. The maximum difference of 0.55 N between the analytical and experimental results was observed on the actuator corresponding to the small fingers when pressurized to 300 kPa.

The results of our experiment indicate that the stability of the actuator during grasping is influenced by the size of the grasped object. Specifically, the bulging effects were reduced as the bending angles increased, particularly when grasping smaller objects. The force estimation results were found to be close to linear when grasping larger objects but became more non-linear as the size of the objects and the length of the actuator increased. It is worth noting that the maximum possible flexion angle of the fingers is 180°, but a flexion angle of 137° is already sufficient for more than 90% of daily functional activities (Hume et al., 1990). Previous studies have reported that normal hand grasping generates fingertip forces ranging from around 0.25 N–3.59 N (Yap et al., 2015). Given these considerations, the output Range-of-Motion and force from the actuators is considered to be sufficient for grasping and gripping most daily items such as bottles and cups.

### Actuator comparison

We present a quantitative comparison between our bi-directional soft actuator and previous designs that control both flexion and extension (Table 1). In our earlier Soft-Elastic Composite Actuator (SECA) design, we used a stiff metal plate to assist with actuator extension upon recoil, which resulted in a rapid response but limited the bending of actuator upon pressurization (Heung et al., 2019a; Heung et al., 2019b). For instance, an ROM of only 68° was reported when the fluid pressure was up to 200 kPa (Heung et al., 2019a). Other designs in the literature used two layers of cavities for independent control of actuator flexion and extension, resulting in similar ROM and output force with less input pressure than our actuator (Yap et al., 2017b; Yap et al., 2017c; Cappello et al., 2018b). For example, the bi-directional actuators required only 70 kPa and 172.4 kPa to achieve ROM of 182° and 120°, respectively, with estimated output forces of 1.58 N and 3 N per actuator. Our actuator requires less pressure than our previous SECA but more than other existing designs due to its smaller size, which is designed to fit human fingers (average finger width and length of 19 and 89 mm, respectively) (Zupko, 1985; Peters et al., 2002). The internal volume used to control both flexion and extension is also reduced, which necessitates a higher pressure to generate comparable ROM and output force. Therefore, when designing an actuator, designers must balance the size of the actuator, the amount of pressure required, and the ROM and output force depending on the needs of individual users.

**TABLE 1:** Comparison of our actuator performance with example fluid-driven bi-directional soft actuators.

Actuators	Working principle	Reported mechanical performance
Soft-elastic composite actuator (Heung et al., 2019a; Heung et al., 2019b)	Silicone rubber-based soft actuator	*Weight:* 37 g
		*Size:* 17 mm (W) × 13 mm (H) × 120 mm (L)
	Flexion is controlled upon pressurization of bladder, and extension is controlled by the metal spring at the bottom of the actuator	*ROM:* 68° at 200 kPa (0.3 mm plate)
		*Force:* 2.45 N at 160 kPa, measured at 0-degree position
Fabric-based bidirectional soft actuator (Yap et al., 2017b; Yap et al., 2017c)	Thermoplastic polyurethane (TPU)-coated fabric-based soft actuator	*Weight:* 110 g (robotic hand)
		*Size:* 25 mm (W) × 25 mm (H) × 190 mm (L)
	Flexion and extension are independently controlled upon pressurization of the internal top and bottom bladders respectively	*ROM:* 182° at 70 kPa
		*Force:* Total 7.9 N at 70 kPa of the robotic hand, measured in the grasp of object with diameter of 50 mm
Knit textile bending actuators (Cappello et al., 2018b)	Thermoplastic elastomer (TPE)-coated fabric-based knit and woven textile actuator	*Weight:* 4.2 g
		*Size:* 30 mm (W) × 30 mm (H) × 120 mm (L)
		*ROM:* 120° at 172.4 kPa
	Flexion and extension are independently controlled upon pressurization of the internal top and bottom bladders respectively	*Force:* Total 15 N at 172.4 kPa of the robotic hand, measured in the grasp of object with diameter of 76 mm
Actuator in this study	Silicone rubber-based soft actuator	*Weight:* 19 g
		*Size:* 12 mm (W) × 12 mm (H) × 105 mm (L)
	Flexion and extension are independently controlled upon pressurization of the internal top and bottom bladders respectively	*ROM:* 172° at 300 kPa
		*Force:* 2.5 N at 300 kPa, measured at 0-degree position

## Preliminary evaluation

### Robotic hand grasping

**FIGURE 9:**
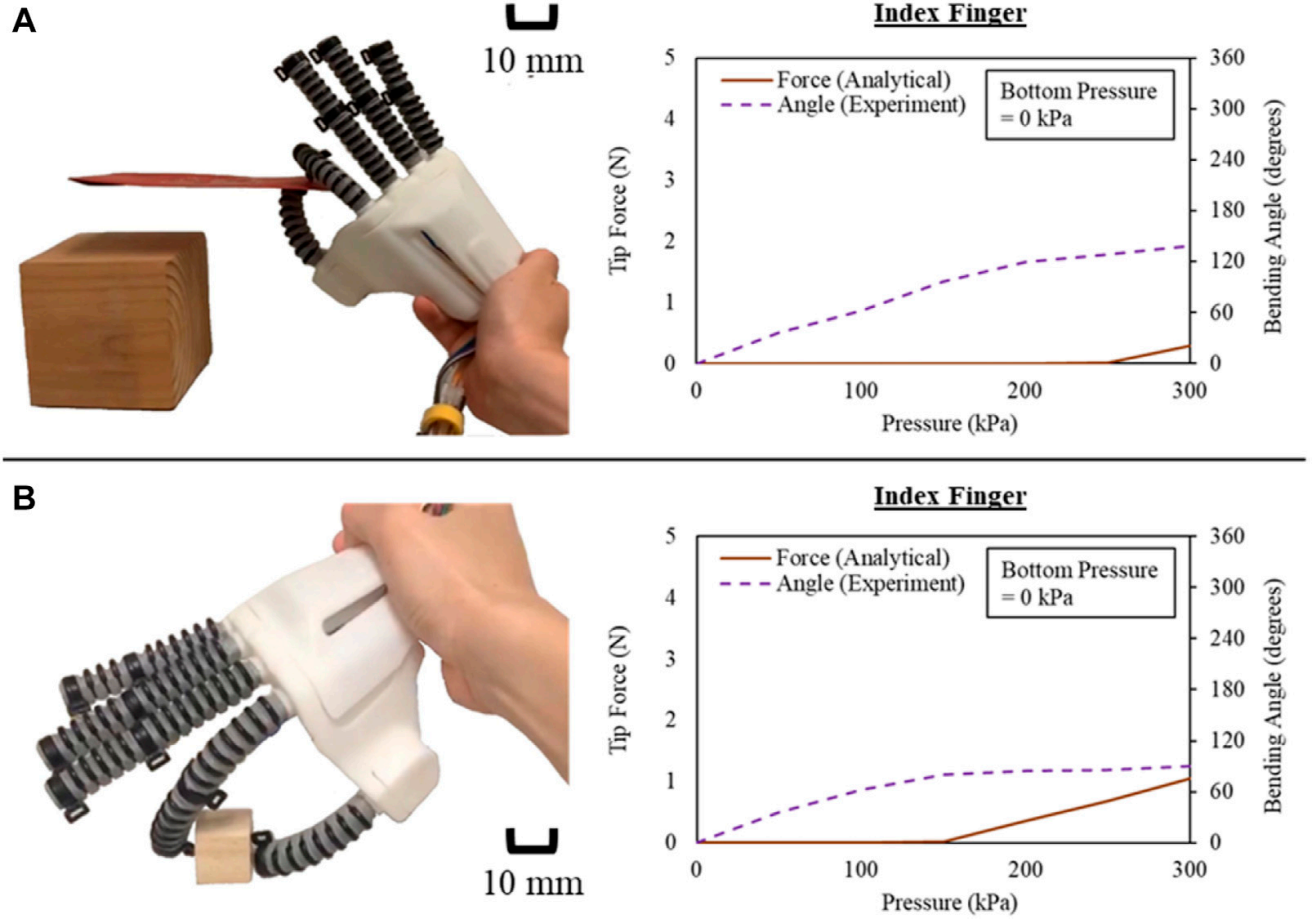
Actuator tip force during the pinch of index finger for **(A)** a card and **(B)** a wooden box.

Before proceeding with human trials, we conducted tests to validate the ability of robotic hand to grasp objects. The purpose of these tests was to evaluate the feasibility of using the soft robotic hand to assist with activities of daily living (ADL). We tested the ability to grasp a card (length of 10 cm and width of 6 cm) and a wooden box (2.5 × 2.5 × 2.5 cm) without being worn on human hands. The bending angle of the actuator corresponding to the index finger was measured by flex sensors upon pressurization. The bending angle was then substituted into mathematical models based on Eqs 13–15, along with the corresponding dimensions of the respective actuator, to estimate the grip force of the soft robotic hand when grasping objects. We manually controlled the soft robotic hand as an indicator to the subject, and a constant pressure of 300 kPa was applied during each actuation step. While the actuator corresponding to the index finger was selected for the evaluation of tip force upon pinching of objects, it was estimated that the pinch force at index finger achieved 0.285 N and 1.05 N for the card and the wooden box respectively at 300 kPa. As a result, the force estimated by the mathematical models of the actuators (Figure 9) was found to be in agreement with previous research conducted for measuring fingertip force during object grasping, within around 1 N–2 N (Yap et al., 2015). This validation test gave us confidence that the soft robotic hand was capable of grasping objects with sufficient force for ADL-related tasks.

### Healthy subjects evaluation

To further evaluate the effectiveness of the soft robotic hand, we recruited two healthy subjects (age: 28, male; age: 26, female) with intact hand function from our research team with informed consent. During the evaluation, the subjects were required to remain relaxed to avoid influencing the bending performance of actuators and ensure optimal estimation of output force.

### Grip force estimation on human fingers

To apply the analytical models based on Eqs 13–15 that estimate the fingertip contact force during the grasp of objects, it is necessary to consider the torque of finger joints that influences the bending of our actuators upon pressurization. Assuming the gap existed between the actuator and the finger to be ignorable, the kinematics of fingers can be represented by.

$$\begin{array}{cc} M_{joint}=k_{joint}\;\left(\theta_{rest}-\theta \right); & \theta <\theta_{rest} \end{array}$$ (16)

where $k_{joint}$ is the finger joint stiffness, θ is the joint angle, and $\theta_{rest}$ is the initial resting angle when there is no exerted voluntary movement, as human fingers tend to curl inwards and remain in a flexed position ( $\theta_{rest}$ ) due to the muscle tone naturally presented in finger flexors (e.g., flexor digitorum profundus) being larger than that of finger extensors (e.g., extensor digitorum) (Wehbé and Hunter, 1985; Loh et al., 2018).

**FIGURE 10:**
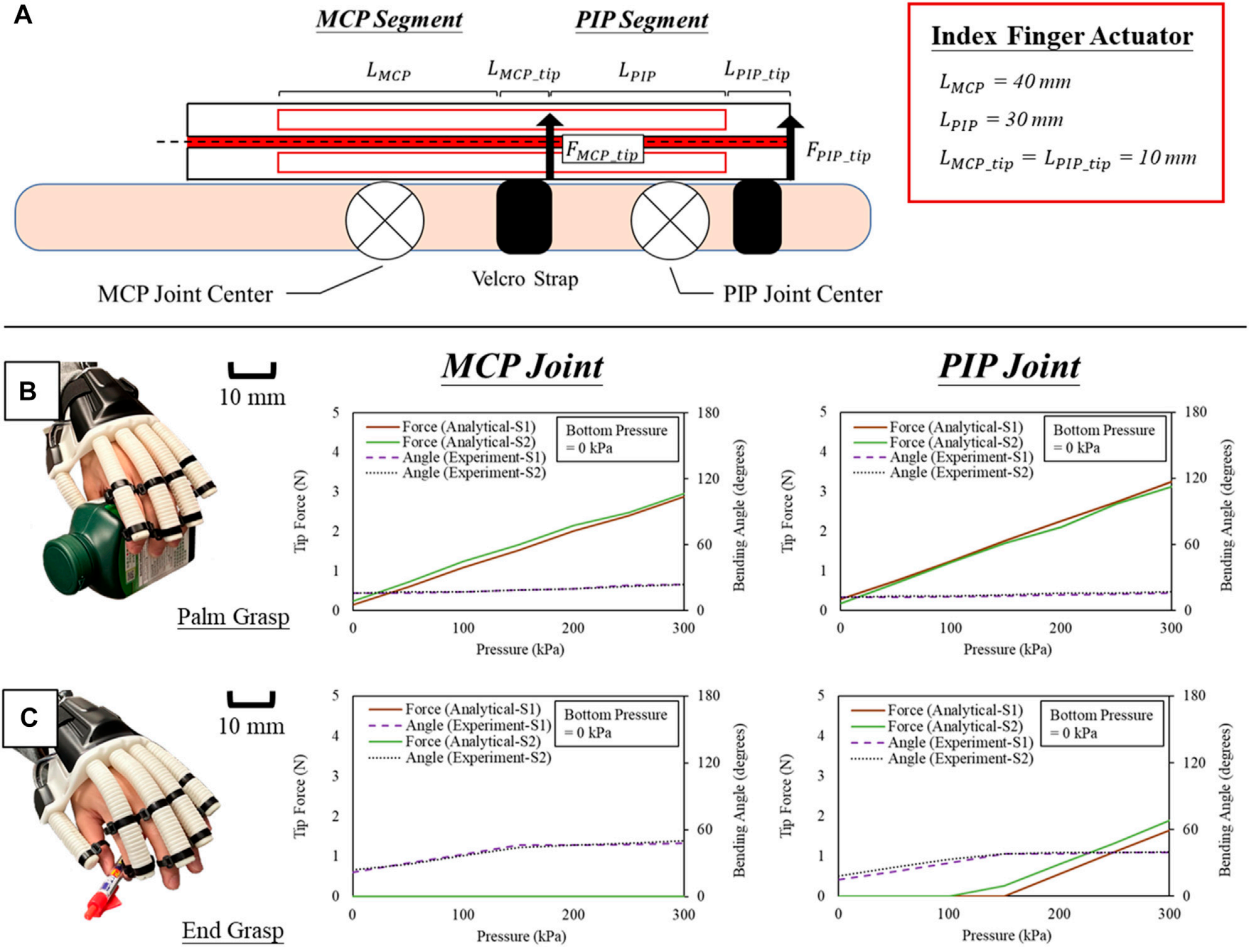
**(A)** Equivalent model of the actuator for analyzing the grip force at MCP and PIP joint respectively, and estimated grip force and measured joint angle during **(B)** palm grasp of a bottle and **(C)** end grasp a pen.

Our bi-directional soft actuator is considered as equivalent to being composed of two segments, i.e., MCP and PIP segments, respectively (Figure 10A). While each segment of our actuator is covering the MCP and PIP joints during the grasp of objects, the bottom cavity inside the actuator remains unpressurized, allowing the actuator kinematics to be given by.

1) MCP Segment when Touching Objects

$$\begin{array}{c} M_{MC{P\_}_{bend}}=M_{\sigma MC{P\_}_{bend}}-M_{\sigma MC{P\_}_{extend}}+M_{MC{P\_}_{sensor}}\\ +M_{MCP\_tip}-M_{MCP\_joint} \end{array}$$ (17)

which.

$$\begin{array}{l} P_{MCP\_bend}=\frac{2}{eb\left(2a+t+b\right)}\\ ∙(\frac{EI\theta }{L_{MCP\_sensor}}+f\left(\theta \right)+F_{MCP\_tip}L_{MCP\_tip}+k_{MCP\_joint}\;\left(\theta_{MCP}-\theta_{MCP\_rest}\right)) \end{array}$$ (18)

and

$$f\left(\theta \right)=M_{\sigma MC{P\_}_{bend}}-M_{\sigma MC{P\_}_{extend}}$$ (19)

2) PIP Segment when Touching Objects

$$\begin{array}{c} M_{PI{P\_}_{bend}}=M_{\sigma PI{P\_}_{bend}}-M_{\sigma PI{P\_}_{extend}}+M_{PI{P\_}_{sensor}}\\ +M_{PIP\_tip}-M_{PIP\_joint} \end{array}$$ (20)

which

$$\begin{array}{c} P_{PIP\_bend}=\frac{2}{eb\left(2a+t+b\right)}\\ ∙(\frac{EI\theta }{L_{PIP\_sensor}}+f\left(\theta \right)+F_{PIP\_tip}L_{PIP\_tip}+k_{PIP\_joint}\;\left(\theta_{PIP}-\theta_{PIP\_rest}\right)) \end{array}$$ (21)

and

$$f\left(\theta \right)=M_{\sigma PI{P\_}_{bend}}-M_{\sigma PI{P\_}_{extend}}$$ (22)

In the experiment, the actuator corresponding to the index finger was selected for the preliminary evaluation of grip force estimation based on the modeling Eqs 17–22. Bending angles of the MCP and PIP segments were measured by flex sensors upon pressurization. The characteristic of index fingers in the two recruited subjects (Table 2) was also obtained for the quasi-static model of MCP and PIP joints presented in Eq. 16, which the joint stiffness was estimated from our previous study in four healthy subjects by averaging the overall results (Heung et al., 2020). Same as the previous section, we manually controlled the robotic hand as an indicator to the subject, and a constant pressure of 300 kPa was applied during each actuation step. We further assigned unilateral tasks involving the palm grasp of a bottle (9 × 7 × 15 cm) and the end grasp of a pen (radius of 1 cm and length of 14 cm) to the subjects, and they successfully performed both tasks while wearing the robotic hand (Figures 10B, C). No active voluntary movement of finger flexion and extension were allowed throughout the whole process. From the results of grasping the bottle, an estimated grip force of 0.15 N and 0.29 N (S1) and 0.25 N and 0.18 N (S2) was naturally presented at both MCP and PIP joint positions prior to robotic hand actuation. They naturally grasped the bottle even without any voluntary movement due to the natural contraction of finger flexors while putting their hands to the bottle. When the maximum pressure of 300 kPa was applied, an estimated grip force of 2.88 N and 2.96 N (S1) and 3.25 N and 3.13 N (S2) was obtained. On the other hand, since the MCP joint was not involved at all during the end grasp, only the grip force at PIP joint position was considered when grasping the pen. When the input pressure reached 100 kPa, the actuator flexed the index finger of S2 touching the pen, and the same for S1 at 150 kPa. A maximum grip force of 1.64 N (S1) and 1.89 N (S2) were estimated at 300 kPa of input pressure. It was noted that the assisted grip force is not constant under the same input pressure and depends on the size of the objects. To grasp an object, smaller size will require a larger actuator bending angle to flex the fingers to the position of the objects, which directly reduces the output force provided for the grasp of smaller objects.

**TABLE 2:** Kinematic characteristics of index fingers.

Subjects	$\theta_{MCP\_rest}$	$\theta_{PIP\_rest}$	$k_{MCP\_joint}$	$k_{PIP\_joint}$
S1 (M*)	46°	40°	0.01876 (Heung et al., 2020)	0.01533 (Heung et al., 2020)
S2 (F*)	58°	49°		

a M: male, F: female.

## Conclusion and future work

In this article, we introduce an optimal bi-directional soft actuator that can actively control both bending and extension while maintaining a size comparable to a normal finger. The actuators are embedded with flexible angle sensors, and we develop an analytical model to quantify the free bending performance and force output of the actuator when pressurized. Experimental results, including bending angles and output force measurements, as well as validation through finite element method (FEM) and analytical results, are presented. Based on the mathematical models, we estimate the output force of the actuators while grasping objects.

To evaluate the grasping ability of the bi-directional soft actuators, we designed and developed a portable soft robotic hand containing five of these actuators. The soft robotic hand is tethered to a portable control system for operation and has shown promise in assisting with activities of daily living, such as grasping. Based on our results, we believe that the portable robotic hand system has the potential to assist patients with hand impairments, such as stroke survivors, in performing ADL-related tasks in various settings, including hospitals, private clinics, or even at home. This demonstration highlights the potential of a soft robotic hand/glove with bi-directional soft actuators in hand rehabilitation. This also has the potential to significantly improve the quality of life for patients with hand impairments by helping them regain some of their independence and perform tasks they may have previously struggled with.

We acknowledge that our models did not fully consider the user intent when voluntarily moving the fingers, e.g., for stroke patients who still have residual ability of performing voluntary movement. Therefore, relying on these models to estimate grip force may result in inaccuracies if the users perform voluntary movement as well during the grasp. These models only focused on complete assistance of hand function without active users’ movement intent and should be considered as preliminary work. To address this limitation, we plan to estimate the level of user intent in finger movement that constantly changing over time based on the iterative learning control method we developed previously to model the voluntary grip force generated by users (Tang et al., 2022). This method will be integrated into the models of bi-directional soft actuators to more accurately estimate the actual grip force experienced by the objects being grasped. More subjects will be recruited to evaluate our robotic hand and we will report the results upon completion of user trial with this advanced model for our bi-directional soft actuator that incorporates finger kinematics and user intents.

## Data Availability

The original contributions presented in the study are included in the article/supplementary material, further inquiries can be directed to the corresponding author.

## References

[B1] Ali A. Fontanari V. Schmoelz W. Fontana M. (2022). Actuator and contact force modeling of an active soft brace for scoliosis. Bioeng. Basel 9 (7), 303. 10.3390/bioengineering9070303 PMC9311770 35877354

[B2] Cappello L. Meyer J. T. Galloway K. C. Peisner J. D. Granberry R. Wagner D. A. (2018a). Assisting hand function after spinal cord injury with a fabric-based soft robotic glove. J. Neuroeng Rehabil. 15 (1), 59. 10.1186/s12984-018-0391-x 29954401 PMC6022347

[B3] Cappello L. Galloway K. C. Sanan S. Wagner D. A. Granberry R. Engelhardt S. (2018b). Exploiting textile mechanical anisotropy for fabric-based pneumatic actuators. Soft Robot. 5 (5), 662–674. 10.1089/soro.2017.0076 30024312

[B4] Connolly F. Walsh C. J. Bertoldi K. (2017). Automatic design of fiber-reinforced soft actuators for trajectory matching. Proc. Natl. Acad. Sci. U. S. A. 114 (1), 51–56. 10.1073/pnas.1615140114 27994133 PMC5224361

[B5] Das R. Babu S. P. M. Visentin F. Palagi S. Mazzolai B. (2023). An earthworm-like modular soft robot for locomotion in multi-terrain environments. Sci. Rep. 13, 1571. 10.1038/s41598-023-28873-w 36709355 PMC9884293

[B6] Dragon Skin Series (). Dragon skin series. Available at: https://www.smoot-hon.com/tb/files/DRAGON_SKIN_SERIES_TB.pdf (accessed March 02, 2023).

[B7] Heung H. Chiu P. W. Y. Li Z. (2016). “Design and prototyping of a soft earthworm-like robot targeted for GI tract inspection,” in Proceeding of the 2016 IEEE International Conference on Robotics and Biomimetics (ROBIO), December 2016 (Qingdao, China: IEEE), 497–502. 10.1109/ROBIO.2016.7866371

[B8] Heung K. H. L. Tong R. K. Y. Lau A. T. H. Li Z. (2019a). Robotic glove with soft-elastic composite actuators for assisting activities of daily living. Soft Robot. 6 (2), 289–304. 10.1089/soro.2017.0125 30874489

[B9] Heung K. H. L. Tang Z. Q. Ho L. Tung M. Li Z. Tong R. K. Y. (2019b). Design of a 3D printed soft robotic hand for stroke rehabilitation and daily activities assistance. IEEE Int. Conf. Rehabil. Robot. 2019, 65–70. 10.1109/ICORR.2019.8779449 31374608

[B10] Heung H. L. Tang Z. Q. Shi X. Q. Tong K. Y. Li Z. (2020). Soft rehabilitation actuator with integrated post-stroke finger spasticity evaluation. Front. Bioeng. Biotechnol. 8, 111. 10.3389/fbioe.2020.00111 32181247 PMC7059754

[B11] Hume M. C. Gellman H. McKellop H. Brumfield R. H. Jr (1990). Functional range of motion of the joints of the hand. J. Hand Surg. Am. 15 (2), 240–243. 10.1016/0363-5023(90)90102-w 2324451

[B12] Jin J. Wang K. Ren L. Qian Z. Lu X. Liang W. (2022). Optimization design of the inner structure for a bioinspired heel pad with distinct cushioning property. Bioeng. (Basel) 10 (1), 49. 10.3390/bioengineering10010049 PMC9854970 36671620

[B13] Leibovic S. J. Bowers W. H. (1994). Anatomy of the proximal interphalangeal joint. Hand Clin. 10 (2), 169–178. 10.1016/s0749-0712(21)01280-4 8040195

[B14] Loh P. Y. Yeoh W. L. Nakashima H. Muraki S. (2018). Deformation of the median nerve at different finger postures and wrist angles. PeerJ 6, e5406. 10.7717/peerj.5406 30123715 PMC6087621

[B15] Majidi C. Kramer R. Wood R. J. (2011). A non-differential elastomer curvature sensor for softer-than-skin electronics. Smart Mat. Struct. 20, 105017. 10.1088/0964-1726/20/10/105017

[B16] Marchese A. D. Katzschmann R. K. Rus D. (2015). A recipe for soft fluidic elastomer robots. Soft Robot. 2 (1), 7–25. 10.1089/soro.2014.0022 27625913 PMC4997626

[B17] Martirosyan N. Kalani M. Y. (2011). Epidermal electronics. World Neurosurg. 76 (6), 485–486. 10.1016/j.wneu.2011.10.001 22251489

[B18] Mohammadi A. Lavranos J. Zhou H. Mutlu R. Alici G. Tan Y. (2020). A practical 3D-printed soft robotic prosthetic hand with multi-articulating capabilities. PLoS One 15 (5), e0232766. 10.1371/journal.pone.0232766 32407396 PMC7224508

[B19] Namdar Ghalati M. H. Ghafarirad H. Suratgar A. A. Zareinejad M. Ahmadi-Pajouh M. A. (2022). Static modeling of soft reinforced bending actuator considering external force constraints. Soft Robot. 9 (4), 776–787. 10.1089/soro.2021.0010 34569882

[B20] Peters M. Mackenzie K. Bryden P. (2002). Finger length and distal finger extent patterns in humans. Am. J. Phys. Anthropol. 117, 209–217. 10.1002/ajpa.10029 11842400

[B21] Polygerinos P. Wang Z. Overvelde J. T. B. Galloway K. C. Wood R. J. Bertoldi K. (2015). Modeling of soft fiber-reinforced bending actuators. IEEE Trans. Robotics 31 (3), 778–789. 10.1109/TRO.2015.2428504

[B22] Raeisinezhad M. Pagliocca N. Koohbor B. Trkov M. (2021). Design optimization of a pneumatic soft robotic actuator using model-based optimization and deep reinforcement learning. Front. Robot. AI 8, 639102. 10.3389/frobt.2021.639102 34026857 PMC8138170

[B23] Rus D. Tolley M. T. (2015). Design, fabrication and control of soft robots. Nature 521 (7553), 467–475. 10.1038/nature14543 26017446

[B24] Sareh S. Noh Y. Li M. Ranzani T. Liu H. Althoefer K. (2015). Macrobend optical sensing for pose measurement in soft robot arms. Smart Mat. Struct. 24, 125024. 10.1088/0964-1726/24/12/125024

[B25] Shi X. Q. Heung H. L. Tang Z. Q. Li Z. Tong K. Y. (2021). Effects of a soft robotic hand for hand rehabilitation in chronic stroke survivors. J. Stroke Cerebrovasc. Dis. 30 (7), 105812. 10.1016/j.jstrokecerebrov-asdis.2021.105812 33895427

[B26] Simone L. K. Kamper D. G. (2005). Design considerations for a wearable monitor to measure finger posture. J. Neuroeng Rehabil. 2 (1), 5. 10.1186/1743-0003-2-5 15740622 PMC555583

[B27] Soft Robotic Toolkit (). Soft robotic toolkit. Available at: https://softroboticstoolkit.com/(accessed March 03, 2023).

[B28] Spark Fun (). Spark fun . https://www.sparkfun.com/datasheets/Sen-sors/Flex/flex22.pdf (accessed March 06, 2023).

[B29] Sparks J. L. Vavalle N. A. Kasting K. E. Long B. Tanaka M. L. Sanger P. A. (2015). Use of silicone materials to simulate tissue biomechanics as related to deep tissue injury. Adv. Skin. Wound Care 28 (2), 59–68. 10.1097/01.ASW.0000460127.47415.6e 25608011

[B30] Tang Z. Q. Heung H. L. Shi X. Q. Tong R. K. Y. Li Z. (2022). Probabilistic model-based learning control of a soft pneumatic glove for hand rehabilitation. IEEE Trans. Biomed. Eng. 69 (2), 1016–1028. 10.1109/TBME.2021.3111891 34516370

[B31] Tong K. Y. Ho S. K. Pang P. K. Hu X. L. Tam W. K. Fung K. L. (2010). An intention driven hand functions task training robotic system. Annu. Int. Conf. IEEE Eng. Med. Biol. Soc. 2010, 3406–3409. 10.1109/IEMBS.2010.5627930 21097247

[B32] Wang Z. Polygerinos P. Overvelde J. T. B. Galloway K. C. Bertoldi K. Walsh C. J. (2017). Interaction forces of soft fiber reinforced bending actuators. IEEE ASME Trans. Mechatron. 22 (2), 717–727. 10.1109/TMECH.2016.2638468

[B33] Wang J. Fei Y. Chen W. (2020). Integration, sensing, and control of a modular soft-rigid pneumatic lower limb exoskeleton. Soft Robot. 7 (2), 140–154. 10.1089/soro.2019.0023 31603736

[B34] Wehbé M. A. Hunter J. M. (1985). Flexor tendon gliding in the hand. Part II. Differential gliding. J. Hand Surg. Am. 10 (4), 575–579. 10.1016/s0363-5023(85)80086-1 4020073

[B35] Xu L. Ge J. Patel J. H. Fok M. P. (2017). Dual-layer orthogonal fiber Bragg grating mesh based soft sensor for 3-dimensional shape sensing. Opt. Express 25 (20), 24727–24734. 10.1364/OE.25.024727 29041418

[B36] Yang M. Liu Q. Naqawe H. S. Fok M. P. (2020). Movement detection in soft robotic gripper using sinusoidally embedded fiber optic sensor. Sensors (Basel). 20 (5), 1312. 10.3390/s20051312 32121229 PMC7085586

[B37] Yap H. K. Lim J. H. Nasrallah F. Goh J. C. H. Yeow R. C. H. (2015). “A soft exoskeleton for hand assistive and rehabilitation application using pneumatic actuators with variable stiffness,” in Proceeding of the IEEE International Conference on Robotics and Automation (ICRA), May 2015 (Seattle, WA, USA: IEEE), 4967–4972. 10.1109/ICRA.2015.7139889

[B38] Yap H. K. Lim J. H. Nasrallah F. Yeow C. H. (2017a). Design and preliminary feasibility study of a soft robotic glove for hand function assistance in stroke survivors. Front. Neurosci. 11, 547. 10.3389/fnins.2017.00547 29062267 PMC5640819

[B39] Yap H. K. Sebastian F. Wiedeman C. Yeow C. H. (2017b). Design and characterization of low-cost fabric-based flat pneumatic actuators for soft assistive glove application. IEEE Int. Conf. Rehabil. Robot. 2017, 1465–1470. 10.1109/ICORR.2017.8009454 28814026

[B40] Yap H. K. Khin P. M. Koh T. H. Sun Y. Liang X. Lim J. H. (2017c). A fully fabric-based bidirectional soft robotic glove for assistance and rehabilitation of hand impaired patients. IEEE Robot. Autom. Lett. 2 (3), 1383–1390. 10.1109/LRA.2017.2669366

[B41] Yu M. Liu W. Zhao J. Hou Y. Hong X. Zhang H. (2022). Modeling and analysis of a composite structure-based soft pneumatic actuators for soft-robotic gripper. Sensors (Basel) 22 (13), 4851. 10.3390/s22134851 35808347 PMC9268967

[B42] Zhang H. Lowe A. Kalra A. Yu Y. (2021). A flexible strain sensor based on embedded ionic liquid. Sensors (Basel) 21 (17), 5760. 10.3390/s21175760 34502651 PMC8434210

[B43] Zupko R. E. (1985). A dictionary of weights and measures for the British isles: The middle ages to the twentieth century. Philadelphia, PA: American Philosophical Society, 109–110.

